# Consumption

**Published:** 1857-04

**Authors:** 


### HALL'S JOURNAL OF HEALTH.

OUR LEGITIMATE SCOPE IS ALMOST BOUNDLESS: FOR WHATEVER BEGETS PLEASURABLE AND HARMLESS FEELINGS, PROMOTES HEALTH; AND WHATEVER INDUCES DISAGREEABLE SENSATIONS, ENGENDERS DISEASE.

VOL. IV.] APRIL, 1857. [NO. IV.

We aim to show how disease may be avoided, and that it is best, when sickness comes, to take no medicine without consulting an educated physician.

### CONSUMPTION

Is a disease which destroys one out of every six grown persons, which invades every family in the civilized world, which carries to the grave, from New York city alone, in the space of a single year, more than three thousand persons. Such a disease ought to be understood, at least as to its general features. Every intelligent individual ought to have a general idea of its nature, its causes, and its prevention. Such a result will be of slow attainment. The thoughtful few will give it attention, which will gradually force itself on the masses, at least so far as the general principles of prevention are concerned.

We believe that, within a few years, there has been a progressive popular intelligence on the subject, and that more correct views prevail in reference to its nature, than at any previous time. Now and then a man of extraordinary intelligence and practical good sense makes his appearance, such as Moreton, of Philadelphia, Wooster, of Cincinnati, and Norcom, of Carolina; but they have passed away, and in a whole lifetime succeeded in impressing their views upon a chance individual here and there.

Two sentiments pervaded the theory and practice of these excellent men—and they were all contemporaries, but most probably wholly unknown to each other. Moretcn was widely known; we never saw the amiable Wooster's name in any printed book; and we ourselves never heard of Norcom until years after his death. They believed that—

1st. No medicine known to them had any uniform appreciable good effect in common consumption of the lungs.

2d. Without a free exposure to out-door air, no case of consumption ever was cured.

These same sentiments are now taking hold of the medical minds of Great Britain, with several remarkable additions: that—

1st. Sea voyages hasten the fatal result of consumptive disease.

2d. Seeking a milder climate precipitates a like result.

3d. The maintenance of an equal artificial temperature only aggravates the disease.

These sentiments are forcibly presented, in scholarly language, by Dr. James E. Pollock, physician to the London Hospital for Consumption, as reported in Braithewaite, part thirty-four, for January, 1857, p. 48, in twenty closely-printed octavo pages. We have seldom seen such a large amount of truthful, practical matter, in any single transient paper. We cordially commend it to professional attention.

We take occasion here to express our high gratification in having such authority to corroborate our views, as presented in our last publication, entitled "*Consumption*," committed to press in October, 1856, while we saw Dr. Pollock's publication, for the first time, in *Braithewaite* for the following January. He agrees with us also in a view which we have taken earnest pains to press on medical attention, to wit: to date the actual commencement of consumption to a point "*higher up*"—we would say *farther back*—than a physical sign of a deposit in the lungs. He says: "There is an antecedent state of disordered health, which, as a causative agent, originates that altered state of the blood which produces tubercle. To this part of our subject, I would entreat your earnest attention. It is not only the key to the disease, but it is the hopeful period for treatment—the critical time in which we may check the inroads of the most fatal of all affections incident to the human frame. Feel no hesitation in saying that the earliest symptom of consumption is 'WASTING;' it precedes cough, and spitting of blood, and hectic, and all the physical signs; * * * hence the second symptom is *debility*." Had Dr. Pollock had our book before him he could not have more thoroughly presented our own views, stating, as we have done, that *deficiency in breath, and strength, and flesh*, are the first far-off symptoms of consumption, coming on as they do long before cough, and spitting of blood, and pains about the chest. We believe that, in the vast majority of cases, the foundation—the first symptoms of consumption in both sexes, is laid at about the age of eighteen or twenty years, and that, if medical attention were secured at this age to children, by which a critical examination could be made as to those points, and corresponding action follow, the mortality from this disease would be largely diminished at once. To neglect these earliest symptoms of consumption, is like neglecting looseness of bowels in Asiatic cholera, which is nothing short of almost certain death.

No one seems to have the slightest fear of consumption now-a-days, unless there is some cough, when the fact is, some persons have observed no cough at all until within three weeks of death, and on examination the lungs have been found a mass of rottenness. In the vast mass of cases, cough is a telegraphic signal that the train has been fired, that tubercles have been deposited, and are taking on irritative and inflammatory action, which is the stage immediately preceding actual decay of the lungs; whereas, if short breath, wasting of flesh, and general weakness, were considered, as they really are, the *avant couriers* of the disease, and corresponding action had, three fourths of those who now die of consumption could ward off the disease indefinitely. Hard-earned thousands, yes millions, often go to distant relations, as heartless as they are greedy, which else would have fallen to natural heirs, if, instead of nursing children from seventeen to twenty years, the moment they seem to become "*weakly*," by consigning them to feather beds, and furnaced rooms, and closed carriages, afraid as death of a single puff of fresh out-door air, lest it should give them a cold—we say a far different result would have followed the hard mattress, the cold sleeping-room, the frequent walk and daily horseback ride, early and late, of from five to thirty miles a day, in spite of all obstacles of wind, and storm, and dust, and snow, and hail—of zero, or of broiling heat.

In this connection, we may not do better than reproduce an article from one of our first numbers, prepared originally for *The Home Journal*, in 1853, by its world-renowned editor, N. P. Willis, who was himself a part of the fact, and lives this day the indisputable evidence of the enduring results of *out-door activities*, as a means of indefinitely postponing the progress of apparent actual Consumption. The whole article might well have been written in italics, for we know of no living writer who can say so much that is true in so few words, as Mr. Willis; we mean personally observed truth—a column of meaning is sometimes embodied in a dozen monosyllables. We have, however, capitalized the sentiment which excels in importance, in our estimation, at least all others:

"THERE WAS NOT A DAY OF THE SUCCEEDING WINTER, HOWEVER COLD OR WET, IN WHICH I DID NOT RIDE EIGHT OR TEN MILES ON HORSEBACK. *With five or six men, I was, for most of the remaining hours of the day, out of doors, laboring at the roads and clearings of my present home.*"

Common consumption of the lungs destroys more people than any other half dozen diseases, while a third of all adults who die in civilized society, do so from ailments connected with the air-passages; hence whatever tends to diffuse true knowledge on the subject must be a public good. Theories are good enough in their place, but the mass of society prefers to deal in facts, in well established, whole facts; these are more tangible, and the common mind can more easily grapple with them. The diseases to which the lungs and their proper appendages are liable, are Asthma, Bronchitis, Consumption, Laryngitis or Throat ail, Croup, Pleurisy, Inflammation of the Lungs, Congestion of the Lungs, Quinsy, &c. All these diseases arise from two causes:

1. Changes of temperature.
2. The failure to keep the breathing apparatus in vigorous, full, healthful operation, by a sufficient amount daily of exertive exercise in the open air. By a wise attention to this second cause of lung diseases, the former will cease to be a cause, except in occasional cases. Not only so, threatened consumption may be effectually warded off, in the vast majority of cases, by the proper adaptation of daily out-door exertive exercise to the requirements of the system, as indicated by the condition of the pulse, the heart, the breathing organs, of which the physician ought to be the standing judge. For, when a man is an invalid, the amount of food, air, and exercise which he needs, requires as much of medical intelligence, experience, and skill, as would the judicious exhibition of medicine.

*An example*—HEART DISEASE.

It is well known that the symptoms of a disease of the heart, and those of the lungs, as well as those of a spinal affection, are so apparently alike in the main, that it requires large medical experience to decide safely and certainly between them; but the exercise requisite in an affection of the lungs would inevitably destroy life if advised for a disease of the heart or spine. In no form of sickness is exercise so immediately and certainly fatal as in heart affections, while the results of active exercise in spinal disease are terrible, literally terrible, not in their immediate effects as involving life, but in the certain penalty of weeks, and months, and weary years of corporeal helplessness, and agonizing, almost ceaseless pain, requiring a thousand times more endurance and a far higher degree of fortitude than marching up to the cannon's mouth in the heat of battle. An affecting instance of this kind came under my notice within a few years past, and I feel sure that a recital of it will be a public benefit, as teaching the importance of taking early competent medical advice in cases of sickness.

On the 23d of September, 1851, I was called to see a young lady on a visit to New York, who was supposed to be in a decline. She was from a neighboring city, an only daughter. She was just entering life, with all the advantages which position, and fortune, and refinement, could bestow. She had a pulse of a hundred and twenty a minute, thirty-six respirations, an incessant cough, debility, such that she could not walk without two assistants. She still lives, a noble monument of heroic endurance and mental energy and worth. Previous to applying to me she had suffered a dozen deaths, in her efforts to take air and exercise on foot, on horse, in carriage; and as often almost as she would take them, she could, on reaching her own door, scarcely prevent herself from shrieking out with an agony of pain. She was encouraged to persevere in these efforts, and, with a daughter's affection for a loving mother, whose solicitude and watchfulness never slept, she did so, until locomotion became impossible. This being a clear case of spinal disease, every step she took, every moment she sat still, aggravated the complaint. The best medical skill in the country has failed to afford her any permanent relief, and to this hour she is unable to stand, suffering daily torture, hardly desiring to hope for even relief until she is called to go where all the good are.

With this case, given as a precaution against the danger which attends taking daily out-door exercise, without medical advice, for any of the prominent symptoms of consumption, such as cough, short breath, quick pulse, debility, pains about the chest, &c., I here give, as being highly instructive, an article from "The Home Journal" of December 10th, 1853, under the editorial head:—

(*For Invalids only.*)

Are you quite well, dear reader? Are all those who are dear to you quite well? If so, perhaps you will kindly pass on to another topic, allowing me, under the Idlewild caption, for this week, to answer a letter from an invalid—the information thus called for being interesting to invalids only, or to those with precious invalids for whom they feel and care. In a world where mortals walk beside Death with a face averted, the sick can talk safely of their sorrows only to the sick. I do not claim, therefore, the attention due to a general topic. Though, with pulmonary consumption for our country's most fatal liability, any experience, in eluding or defeating it, may be of interest to so many, as to be, at least, excusably tedious to the remainder. It comes appropriately from Idlewild. The Highlands around us, I fully believe, are the nearest spot to New York, where the acrid irritation of our eastern and seaboard climate is unfelt. Poke your fire, then, dear delicate reader!—(for you are an invalid, by your following me thus far)—and settle yourself comfortably in your arm-chair, while I lay before you a sad and well-written letter from an invalid:

"*C*******, *November* 21, 1853.

"MR. WILLIS: *Dear sir*,—You will perhaps think it presumption in me, an entire stranger, to address you as I now do; but I shall be willing to abide your judgment after you have heard my story. I am a Presbyterian clergyman, in feeble health. After five years' preaching in one happy parish, my lungs gave out, and I was obliged to give up my calling. By the advice of physicians here and in New York, I spent two winters at the South, roaming from place to place, but spending most of the time in Jacksonville and St. Augustine, Florida. I was there during the winter of your tour in that region, and on the same sad errand. And I may here say, that I have taken great pleasure in reading, weekly, your record of travel in those parts.

"But I got no essential benefit from the 'Sunny South'—nothing but some disgust for it, weariness of travel and a warmer love for the North and for my home. Neglecting further medical advice, I bought, two years since, a pleasant site for a country residence in this, my native place, built a house, and devoted myself to tree-planting and gardening of all sorts. This has been my sole employment for two summers. In winter I warm my whole house *moderately*, not allowing the mercury to rise above sixty or sixty-two degrees, and connect with this a thorough ventilation. I remain within doors most of the time. Between romping with my two children, playing with grace-sticks, battle-door, etc., fighting imaginary foes with my cane, and the music of a piano, I manage to get regular, daily exercise and recreation. In favorable weather I also take a brisk walk of half a mile.

" This mode of life makes me quite happy, and I enjoy a tolerable degree of health ; but *I don't get well.* I followed you to Idlewild with much interest, having a fellow-feeling on one point, at least, and watched to see whether you would get the mastery of disease. In your last letter you say that you are no longer to be classed among consumptives. Alas! I can't say as much for myself, I fear. And on reading your lines, I resolved to write to you, as a once fellow-invalid, and ask, *What has cured you?* The doctors advise me to go South and take cod-liver oil, but their prescriptions do me no good, and I improve most when following my own judgment. I spade, and hoe, and rake quite lustily, and ride horseback, in summer; I cough but little, and eat and sleep as well as ever—but cannot use my lungs. Now, may I trouble you to give me some plain advice—a little of your own daily regimen—if you are willing to do so, an account of what has helped you.

" I consult you, not as a doctor, but as a man of benevolence, knowing by experience the feelings of a young man arrested by disease, and laid aside from the activities of life.

" If you do not think proper, or find it convenient, to address me personally, I beg leave to suggest that you give your friends, through the *Home Journal*, some of your views and your experience relating to the treatment of pulmonary affections. A large and eagerly attentive audience would listen to your words, I assure you.

" Pardon me, sir, if I have annoyed you by this letter ; and if you are willing to do so, please allow me to hear from you, and greatly oblige, yours, with true respect, A. D. G."

[To which straightforward and touching letter, the following was the bulk of my reply—not very satisfactory, I fear, though possibly there may be a point or so in which it is either suggestive or corroborative :]—

* * The politicians teach us how to treat a disease, I think. They do not try to *convert* the opposing party. They are content if they can *keep it in the minority*—sure that it will tire, in time, of its want of power, change sides, or disappear. The patient who troubles himself least about his disease, (or leaves it entirely to his doctor,) but who perseveringly *outvotes it* by the high condition of the *other parts* of his system, is the likeliest to recover—and it is of this *high condition*, alone, that I have anything to say. Of twenty who may be sleepless with a cough and weakened with the raising of blood, no two, perhaps, are subjects for precisely the same medical treatment, or diseased in precisely the same locality, though all are called "consumptives." Our friends, the physicians, are better geographers than we, as to where the healing is wanted, though they strangely confine themselves to the specific ailment, taking it for granted that the patient keeps the rest of his body in proper training for recovery. It is medical etiquette, I believe, to refrain from any very particular inquiry into this. But few sick men are wise or firm-minded enough to be safely trusted with their own general condition; and I, for one, came very near dying—not of my disease, but of what my doctors took for granted.

To leave generalities, however, and come to the personal experience which you ask for:

I went to the Tropics, as a last hope, to cure a chronic cough and blood-raising which had brought me to the borders of the grave. I found a climate in which it is hard to be unhappy about anything—charming to live at all—easy to die. (At least, those who were sure of dying, and did die—and in whose inseparable company I thought I was—were social and joyous to the last.) The atmosphere of that Eden-latitude, however, is but a pain-stilling opiate, while the Equator might be called a kitchen-range for a Sardanapalus, and the Antilles are but tables loaded with luxuries. The Caribbean Sea is the Kingdom of the Present Moment. The Past and the Future are its Arctic and Antarctic—unthought of, except by desperate explorers. Hither are sent invalids, with weakened resolution, to make a pilgrimage with prescription and prudence! You may see by the book I have just published, (Health-Trip to the Tropics,) with what complete forgetfulness of care or caution I made one of an invalid company for months. Was anybody going to be shut up in a bedroom with such nights out of doors? Was anybody going to be dull and abstinent with such merry people, and a French breakfast or tempting dinner on the table?

I reached home in July, thoroughly prostrated, and, in the opinion of one or two physicians, a hopeless case. Coughing almost the whole of every night, and raising blood as fast as my system could make it, I had no rest and no strength. I lingered through the summer, and, as the autumn came on, and the winter was to be faced, I sat down and took a fair look at the probabilities. With the details of this troubled council of war, I will not detain you; but, after an unflinching self-examination,

I came to the conclusion that I was, myself, the careless and indolent neutralizer of the medicines which had failed to cure me—that one wrong morsel of food or one day's partially neglected exercise might put back a week's healing—and that, by slight omissions of attention, occasional breaking of regimen, and much too effeminate habits, I was untrue to the trust which Gray, my friend and physician, had made the ground of his prescriptions. And, to a minutely persevering change in these comparative trifles, I owe, I believe, my restoration to health. There was not a day of the succeeding winter, however cold or wet, in which I did not ride eight or ten miles on horseback. With five or six men, I was, for most of the remaining hours of the day, out of doors, laboring at the roads and clearings of my present home. The cottage of Idlewild was then unbuilt, and the neighboring farm-house, where we boarded, was, of course, indifferently warmed; but, by suffering no state of the thermometer to interrupt the morning cold bath, and the previous friction with flesh-brushes, which makes the water as agreeable as in summer, I soon became comparatively independent of the temperature *in* doors, as my horse and axe made me independent of it when *out* of doors. With proper clothing to resist cold or wet, I found (to my surprise) that there was no such thing as disagreeable weather to be felt in the saddle; and, when a drive in a wagon or carriage would have intolerably irritated my cough, I could be all day in the woods with an axe, my lungs as quiet as a child's.

With all this, and looking like the ruddiest specimen of health in the country round about, I am still (you will be comforted to hear) troubled occasionally with my sleep-robber of a cough; and in Boston, the other day, on breathing that essence of pepper and icicles which they call their "East Wind," I was seized with the old hemorrhage of the lungs, and bled myself weak again. But I rallied immediately on returning to this Highland air, and am well once more—as well, that is to say, as is consistent with desirable nervous susceptibility. The kiss of the delicious South Wind of to-day, (November 30,) would be half lost upon the cheek of perfect health.

I fear I cannot sufficiently convey to you my sense of the importance of *a horse* to an invalid. In my well-weighed opinion, ten miles a day in the saddle would cure more desperate cases, (particularly of consumption,) than all the changes of climate and all the medicines in the world. It is vigorous exercise without fatigue. The peculiar motion effectually prevents all irritation of cold air to the lungs, on the wintriest day. The torpid liver and other internal organs are more shaken up and vivified by the trot of a mile than by a week of feeble walking. The horse (and you should own and love him) is company enough, and not too much. Your spirits are irresistibly enlivened by the change of movement and the control of the animal. Your sense of strength and activity, (in which lies half the self-confidence as to getting well, which the doctors think so important,) is plus one horse. With the difference from walking, as to pulling upon the forces of the spine and consequently upon the brain, it is recommended by the best English physicians as much the preferable exercise for men of intellectual pursuits. And, last, (I think not least,) the lungs of both body and soul are expanded by the daily consciousness of inhabiting a larger space —by having an eagle's range rather than a snail's—by living a life which occupies ten miles square of the earth's surface, rather than that " half mile" which you speak of as the extent of your daily walk. The cost is trifling. At this particular season, when horses are beginning, as they say at the livery stables, to " eat their heads off," you may buy the best you can want for fifty dollars, and his feed costs thirty cents a day. As the horse and the doctor are seldom necessities of one and the same man, you may rather find it an economy—apothecary and all.

In that " majority" I have spoken of above, there are (as in all majorities) some voters of not much consequence individually, but still worth keeping an eye upon. Briefly to name one or two:—There are so few invalids who are invariably and conscientiously *untemptable* by those deadly domestic enemies, *sweetmeats*, *pastry* and *gravies*, that the usual civilities at a meal are very like being politely assisted to the grave. The care and nurture of the *skin* is a matter worth some study; for it is capable not only of being negatively healthy, but positively luxurious in its action and sensations—as every well-groomed horse knows better than most men. The American *liver* has a hard struggle against the greasy cookery of our happy country. The impoverished *blood* of the invalid sometimes requires that "glass of wine for the stomach's sake" recommended by the Apostle. Just *sleep* enough and just *clothing* enough are important adjustments, requiring more thought and care than are usually given to them. For a little philosophy in your habitual *posture* as you sit in your chair, your *lungs* would be very much obliged to you. An analysis of the *air* we live and sleep in, would be well worth looking into occasionally. And there are two things that turn sour in a man, without constant and sufficient occupation upon something besides the domestic circle—the *temper* and the *ambition*. * *

Thus much, of my reply to our clerical fellow-sufferer, may interest you, dear invalid reader. Of the medicine of " Out-doors at Idlewild"—the mingled salubrity of the climate of mountain and river around us—I should have said more to one unanchored in a home and a parish. From one who writes so frankly and sensibly as he, we must hope to hear again, however, and, with another opportunity, I may again ask for invalid indulgence, and return to the theme.

While this article embraces the main principles of cure for actual consumption, we are anxious to draw attention to far earlier symptoms of the disease, when its eradication may be made a pleasure rather than a pain—a certainty rather than a mere possibility—a matter of scientific calculation rather than vague theory or opinion—a thing of absolute measurement rather than indefinite conjecture. This is done by rather a new name in medical literature, SPIROMETRY—which means, literally, "*Breath Measurement*"—which enables us to ascertain, with mathematical accuracy and certainty, down to a single cubic inch, what amount of lungs are in efficient operation in any given case; and by knowing what amount each individual should have, varying as to age, sex, &c., according to ascertained laws, we are able, in all cases, to ascertain precisely the extent of the malady. And this is of great practical importance; for if all of a man's lungs are within him, and in free operation, the idea of actual consumption is utterly groundless. On the other hand, if a man, who should have two hundred and fifty cubic inches of air in sound health, has half of his lungs eaten away by consumption, he can no more measure over one hundred and twenty-five than he could fly; and so of any other proportion. But these are fully entered into in our book on "*Consumption.*"

---

